# Do accurate HIV and antiretroviral therapy knowledge, and previous testing experiences increase the uptake of HIV voluntary counselling and testing? Results from a cohort study in rural Tanzania

**DOI:** 10.1186/1471-2458-13-802

**Published:** 2013-09-04

**Authors:** Annabelle South, Alison Wringe, Yusufu Kumogola, Raphael Isingo, Rose Manyalla, Caoimhe Cawley, Basia Zaba, Jim Todd, Mark Urassa

**Affiliations:** 1 Faculty of Epidemiology and Population Health, London School of Hygiene and Tropical Medicine, London, UK; 2 National Institute for Medical Research, Mwanza, United Republic of Tanzania

**Keywords:** HIV, VCT, HIV testing, Tanzania, Cohort study

## Abstract

**Background:**

Despite the introduction of free antiretroviral therapy (ART), the use of voluntary counselling and testing (VCT) services remains persistently low in many African countries. This study investigates how prior experience of HIV and VCT, and knowledge about HIV and ART influence VCT use in rural Tanzania.

**Methods:**

In 2006–7, VCT was offered to study participants during the fifth survey round of an HIV community cohort study that includes HIV testing for research purposes without results disclosure, and a questionnaire covering knowledge, attitudes and practices around HIV infection and HIV services. Categorical variables were created for HIV knowledge and ART knowledge, with “good” HIV and ART knowledge defined as correctly answering at least 4/6 and 5/7 questions about HIV and ART respectively. Experience of HIV was defined as knowing people living with HIV, or having died from AIDS. Logistic regression methods were used to assess how HIV and ART knowledge, and prior experiences of HIV and VCT were associated with VCT uptake, with adjustment for HIV status and socio-demographic confounders.

**Results:**

2,695/3,886 (69%) men and 2,708/5,575 women (49%) had “good” HIV knowledge, while 613/3,886 (16%) men and 585/5575 (10%) women had “good” ART knowledge. Misconceptions about HIV transmission were common, including through kissing (55% of women, 43% of men), or mosquito bites (42% of women, 34% of men).

19% of men and 16% of women used VCT during the survey. After controlling for HIV status and socio-demographic factors, the odds of VCT use were lower among those with poor HIV knowledge (aOR = 0.5; p = 0.01 for men and aOR = 0.6; p < 0.01 for women) and poor ART knowledge (aOR = 0.8; p = 0.06 for men, aOR = 0.8; p < 0.01 for women), and higher among those with HIV experience (aOR = 1.3 for men and aOR = 1.6 for women, p < 0.01) and positive prior VCT experience (aOR = 2.0 for all men and aOR = 2.0 for HIV-negative women only, p < 0.001).

**Conclusions:**

Two years after the introduction of free ART in this setting, misconceptions regarding HIV transmission remain rife and knowledge regarding treatment is worryingly poor, especially among women and HIV-positive people. Further HIV-related information, education and communication activities are urgently needed to improve VCT uptake in rural Tanzania.

## Background

Voluntary counselling and testing (VCT) for HIV, which is initiated by clients wanting to learn their status, is an important part of the continuum of HIV care. VCT serves as a gateway to treatment [1] and may help individuals to protect themselves or their partners from HIV [2,3], as well as enabling them to plan for the future in the light of their status.

The number of facilities offering VCT in sub-Saharan Africa more than trebled between December 2007 [1] and 2011 [4]. Despite this, uptake of VCT remains persistently low, with Demographic and Health Survey (DHS) data from 2010 showing that only 55% of women and 40% of men in Tanzania had ever undergone a HIV test and had received the results, while 30% of women and 25% of men had done so in the 12 months preceding the survey [5].

It was predicted that the rollout of ART services in sub-Saharan Africa would lead to rapid increases in the uptake of VCT, as people would be motivated to learn their status in order to access treatment [6]. However, qualitative research from Mwanza region in rural Tanzania has shown that misconceptions around ART are common, with some people believing that ART kills people [7,8], or that HIV can be cured by traditional healers [9] or miracles [10]. These beliefs may mean that people who suspect that they are infected are less likely to take up VCT services, if they do not expect that it would lead to an effective treatment for their condition.

Further research from the same setting has also shown that although ART availability has led to a “process of normalisation” and decreased internalised stigma [11], the positive health effects of ART have created new sources of stigma, as communities worry that the improved health of people living with HIV may mean they are more likely to have sex and transmit the virus [12]. These new sources of stigma have contributed to a situation of collective denial where alternative, traditional beliefs about disease aetiology provide a less stigmatised explanation for HIV symptoms, partially explaining the relatively slow increase in the rate of VCT uptake in this setting following the introduction of ART [12].

Indeed, misconceptions surrounding the aetiology of HIV infection are rife in rural Tanzania. The belief that HIV or AIDS is caused by bad luck or witchcraft is common [9,12,13], and is often linked to treatment-seeking from traditional healers [7]. Beliefs that the Devil could “take the form of HIV”, creating a ‘false’ virus, which can be cured by prayers have also been noted in the same area [10]. Other misconceptions about HIV transmission include touching or sharing food with a person with AIDS, or via mosquitoes (13). It is likely that the type of person who has not acquired correct knowledge about HIV, despite living in a community seriously affected by the disease, may find it more challenging to learn their HIV status. However, the extent to which such widespread misconceptions regarding modes of HIV transmission may have a detrimental effect on the subsequent use of HIV testing services has not been previously assessed.

Several quantitative studies in sub-Saharan Africa have investigated how socio-demographic, behavioural and health status factors are associated with uptake of VCT services, and have identified differing effects across settings [14]. However, to date, few of these studies have examined how knowledge of HIV, ART and experience of HIV and VCT may also influence service uptake in the era of greater access to HIV treatment. Understanding whether these factors are associated with VCT uptake may help to inform decisions on how best to invest resources to increase the use of HIV testing services.

## Methods

### Study site

Kisesa ward is located in north-west Tanzania, approximately 20 km along the main road that links Mwanza to the Kenyan border. The ward has a population of just under 30,000 people residing in three remote rural villages, three roadside villages and the central trading centre. Income per capita is below US$120 a year, with most economic activities revolving around subsistence farming and petty trading. HIV prevalence in 2006–7 was 6% for men and women [15].

A community-level HIV open cohort study has been monitoring the dynamics of the epidemic in relation to mortality, sexual behaviour and use of health services since 1994. Demographic information is collected during bi-annual household visits, and linked to data obtained during serological surveys that are carried out at a central point in each village approximately every three years. Participants are eligible for the serosurvey if they were resident in the ward during the preceding demographic survey. Eligible individuals are invited to a central point in the village to be interviewed and provide a blood sample [16]. The serological surveillance rounds include HIV testing for research purposes without disclosure of results and a questionnaire that covers socio-demographic characteristics, HIV knowledge and experience, use of health services including VCT, and since 2006–7, knowledge about ART. This study uses data from the fifth serosurvey, carried out in 2006–7.

Since the third serosurvey round, participants have been offered a separate VCT service if they wished to learn their HIV status, with rapid HIV tests available by the fifth round enabling results to be provided within 15 minutes of testing. Data on VCT uptake during the survey are linked to the main cohort dataset using anonymous unique identifiers. VCT has also been available to study participants at a clinic located within the local health centre in the trading centre since 2005. Individuals who were diagnosed with HIV at either the permanent VCT clinic, or at the temporary village-based VCT services were referred to the zonal referral hospital in Mwanza, which began providing free ART in early 2005 [7] before decentralisation of HIV treatment to the Kisesa Health Centre from 2008.

### Data analysis

Data analysis was performed using Stata 10.1 (Stata Corporation, College Station, TX, USA). HIV knowledge, experience, VCT experience and ART knowledge were assessed through several questions (Tables 1 and 2). A composite categorical indicator was then created for each of the four topics. Good HIV knowledge was defined as answering at least 4 out of 6 questions about HIV correctly, fair knowledge was defined as correctly answering 2–3 questions correctly, and poor knowledge was defined as correctly answering 1 or no questions correctly. Good ART knowledge was defined as answering at least 5 out of 7 questions about ART correctly, fair knowledge was correctly answering between 2–4 questions about ART correctly, and poor knowledge was answering only one or no questions correctly. Participants were defined as having experience of HIV if they answered affirmatively to at least one of four questions about whether they knew someone in the village living with HIV, or who had died of AIDS, or had a relative living with HIV or who had died of AIDS. Good VCT experience was defined as having previously had VCT, and answering 4–5 questions about the experience positively, poor VCT experience was defined as having previously had VCT and answering 0–3 questions positively about the experience, and no VCT experience was defined as not having had VCT previously.

**Table 1:** **Baseline characteristics and VCT use by sex**^1^

**Variable**	**Males**				**Females**			
	**Total**		**Used VCT**		**Total**		**Used VCT**	
	**N**	**(%)**	**n**	**(%)**	**N**	**(%)**	**n**	**(%)**
**All**	3, 886	(41)	749	(19)	5, 575	(59)	896	(16)
**HIV knowledge**								
Good	2, 695	(69)	597	(22)	2, 708	(49)	556	(21)
Fair	926	(24)	132	(14)	2, 169	(39)	284	(13)
Poor	265	(7)	20	(8)	698	(13)	56	(8)
**ART knowledge**								
Good	613	(16)	153	(25)	585	(10)	132	(23)
Fair	2, 361	(61)	488	(21)	2, 880	(52)	543	(19)
Poor	912	(23)	108	(12)	2, 110	(38)	221	(10)
**Experience**								
Yes	1, 970	(51)	460	(23)	2, 494	(45)	537	(22)
No	1, 916	(49)	289	(15)	3, 081	(55)	359	(12)
**VCT experience**								
Good	477	(12)	175	(37)	470	(8)	145	(31)
Poor	78	(2)	22	(28)	154	(3)	45	(29)
None	3, 327	(86)	552	(17)	4, 946	(89)	706	(14)
**HIV status**								
HIV negative	3, 680	(95)	687	(19)	5, 156	(93)	806	(16)
HIV positive	192	(5)	60	(31)	389	(7)	87	(22)
**Age years**								
15-24	1, 698	(44)	233	(14)	1, 929	(35)	299	(16)
25-34	764	(20)	223	(29)	1, 409	(25)	304	(22)
35-44	521	(13)	136	(26)	860	(15)	170	(20)
45-54	378	(10)	88	(23)	584	(10)	89	(15)
55+	516	(13)	69	(13)	776	(14)	33	(4)
**Education level**								
None	704	(18)	97	(14)	2, 152	(39)	245	(11)
Primary 1-4	490	(13)	76	(16)	478	(9)	78	(16)
Primary 5-7	2, 096	(54)	423	(20)	2, 509	(45)	476	(19)
Secondary	592	(15)	153	(26)	431	(8)	97	(23)
**Residence**								
Rural	2, 249	(60)	450	(20)	3, 340	(62)	510	(15)
Roadside	799	(21)	152	(19)	1, 607	(20)	161	(15)
Trading centre	701	(19)	124	(18)	962	(18)	186	(19)
**Marital status**								
Ever married	2, 143	(65)	499	(23)	4, 429	(90)	752	(17)
Never married	1, 140	(35)	214	(19)	510	(10)	105	(21)

^**1**^All percentages in this table are column percentages for each variable, except the first row of the table, which gives the percentage of all respondents (i.e. 41% of all respondents were male, 19% of males used VCT).

**Table 2:** **HIV knowledge and ART knowledge by sex and HIV status**^**1**^

**HIV knowledge by sex and HIV status**									
**Variable**	**Total**	**Male**		**Female**		**HIV negative**		**HIV positive**	
		**(n)**	**(%)**	**n**	**(%)**	**n**	**(%)**	**n**	**(%)**
**Total**	9, 461	3, 881	(41)	5, 575	(59)	8, 848	(94)	581	(6)
Ever heard about HIV?									
Yes	9, 236	3, 817	(98)	5, 419	(97)	8, 622	(98)	577	(99)
No	213	64	(2)	149	(3)	209	(2)	4	(1)
Can correctly name 3 or modes of transmission									
Yes	3, 519	1, 958	(50)	1, 561	(28)	3, 272	(37)	234	(40)
No	5, 942	1, 928	(50)	4, 014	(72)	5, 576	(63)	347	(60)
Know healthy looking people can have HIV?									
Yes	6, 527	2, 862	(75)	3, 665	(68)	6, 071	(70)	429	(74)
No	2, 712	956	(25)	1, 756	(32)	2, 554	(30)	128	(26)
Know mosquito bites cannot spread AIDS									
Yes	5, 665	2, 508	(66)	3, 157	(58)	5, 316	(62)	331	(57)
No	3, 574	1, 310	(34)	2, 264	(42)	3, 309	(38)	246	(43)
Know AIDS cannot be transmitted by sharing cups and plates									
Yes	6, 286	2, 719	(71)	3, 567	(66)	5, 874	(68)	397	(69)
No	2, 953	1, 099	(29)	1, 854	(34)	2, 751	(32)	180	(31)
Know AIDS cannot be transmitted by kissing									
Yes	4, 607	2, 185	(57)	2, 422	(45)	4, 307	(50)	286	(50)
No	4, 632	1, 633	(43)	2, 999	(55)	4, 318	(50)	291	(50)
HIV knowledge (composite indicator)									
Good	5, 403	2, 695	(69)	2, 708	(49)	5, 046	(57)	341	(59)
Fair	3, 095	926	(24)	2, 169	(39)	2, 891	(33)	191	(33)
Poor	963	265	(7)	698	(13)	911	(10)	91	(8)
**ART knowledge by sex and HIV status**									
Total	9, 641	3, 881	(41)	5, 575	(59)	8, 848	(94)	581	(6)
Know someone taking ART									
Yes	1, 471	655	(17)	816	(15)	1, 349	(15)	116	(20)
No	7, 977	3, 225	(83)	4, 752	(85)	7, 481	(85)	465	(80)
Has correct knowledge of where ART is available locally									
Yes	2, 968	1, 437	(37)	1, 531	(27)	2, 731	(31)	228	(39)
No	6, 493	2, 449	(63)	4, 044	(73)	6, 117	(69)	353	(61)
Knows ART can only slow down HIV, not stop it									
Yes	7, 060	3, 240	(83)	3, 820	(69)	6, 582	(75)	455	(78)
No	2, 392	642	(17)	1, 750	(31)	2, 252	(25)	126	(22)
Does not think ART drugs are dangerous and can kill people									
Yes	3, 137	1, 503	(39)	1, 634	(29)	2, 922	(33)	204	(35)
No	6, 315	2, 379	(61)	3, 936	(71)	5, 912	(67)	377	(65)
Knows ART drugs have to be useful for life									
Yes	4, 103	2, 082	(54)	2, 021	(36)	3, 810	(43)	278	(48)
No	5, 346	1, 799	(46)	3, 547	(64)	5, 021	(57)	303	(52)
Knows ART drugs are available free of charge in Tanzania									
Yes	4, 146	1, 936	(50)	2, 210	(40)	3, 831	(43)	298	(51)
No	5, 305	1, 946	(50)	3, 359	(60)	5, 002	(57)	283	(49)
Knows that not everyone who is infected needs ART									
Yes	731	261	(7)	470	(8)	681	(8)	48	(8)
No	8, 715	3, 618	(93)	5, 097	(92)	8, 148	(92)	532	(92)
Knowledge of ART (composite indicator)									
Good	1, 198	613	(16)	585	(10)	1, 089	(12)	103	(18)
Fair	5, 241	2, 361	(61)	2, 880	(52)	4, 900	(55)	325	(56)
Poor	3, 022	912	(23)	2, 110	(38)	2, 859	(32)	153	(26)

^1^All percentages in this table are column percentages for each variable except the first row of the table which gives the percentage of all (i.e. 41% respondents were male, 94% were HIV negatives).

The variables assessing HIV knowledge, HIV experience, VCT experience and ART knowledge were described by sex and HIV status. VCT use at the serosurvey was then cross-tabulated by the composite HIV knowledge, HIV experience, VCT experience and ART knowledge variables, and by HIV status, age, education, residence and marital status. This was done separately for males and females, as sex was considered an *a priori* effect modifier. Pearson’s chi-squared odds ratios and Mantel-Haenszel stratum-specific odds ratios were examined where it was hypothesised that a particular variable may modify the effect of the exposure variable of interest on the outcome.

Exposure variables, potential confounders and interaction terms that were found to be significantly associated with VCT uptake in crude and stratified analysis were forward-fitted to a logistic regression model to calculate adjusted odds ratios. The likelihood ratio test was used to test the overall effect of each variable in the model, and those that significantly (p < 0.05) improved the fit of the model were retained. The Wald test was used to test the significance of the odds ratios at each level within the variable.

### Ethics

The Tanzanian Medical Research Coordinating Committee and the London School of Hygiene and Tropical Medicine Ethics Committee approved the study.

## Results

### Characteristics of the participants

3,886 men and 5,575 women participated in the survey, corresponding to 61% of the eligible population [15]. 82% of men and 61% of women were educated to at least primary school level and 59% of participants lived in rural villages. Additional baseline characteristics of the population are shown in Table 1.

### Knowledge of HIV and ART

Table 2 shows the distribution of HIV knowledge by sex and HIV status. Nearly all participants had heard about HIV. Men had better knowledge about HIV than women, with 69% of men having good HIV knowledge compared to 49% of women. HIV-positive males had the best knowledge about HIV. Although 88% of men and 72% of women reported knowing how HIV was transmitted, only 50% of men and 28% of women could correctly name three or more modes of transmission. Women were more likely than men to believe common misconceptions about HIV, including transmission of HIV through kissing (55% of women versus 43% of men), or by mosquito bites (42% of women and 34% of men).

Few people (17% of men and 15% of women) reported knowing someone who was taking ART, with males and HIV-positive people most likely to report this. A higher proportion of men than women were able to correctly identify where ART was available (37% versus 27%). More than half of participants said that ART is dangerous and can kill people (61% of men and 71% of women). Less than half of participants knew that ART was available free of charge in Tanzania (50% of men, 40% of women, 51% of HIV-positive people, 43% of HIV-negative people). Overall, 16% of men and 10% of women had “good” knowledge of ART.

**Figure 1:**
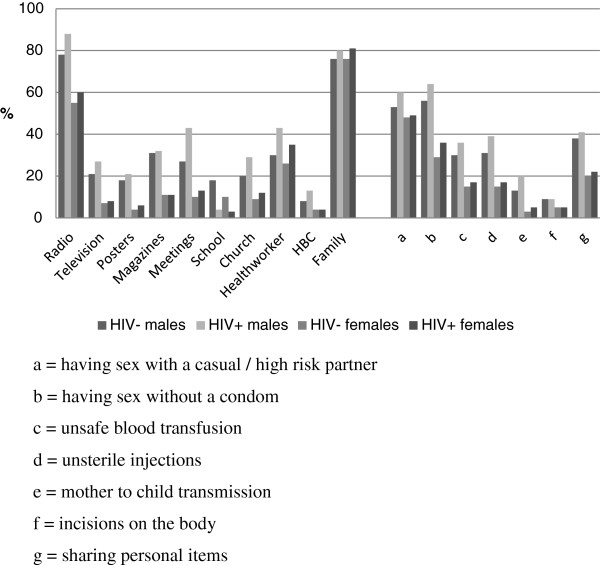
Sources of information about HIV and knowledge of modes of transmission, by sex and HIV status.

Figure 1 shows the percentage of people reporting different sources of knowledge about HIV by sex and HIV status. The most commonly reported source of knowledge about HIV was family, which was reported by almost 80% of participants regardless of sex or HIV status. Radio was the next most commonly reported source, with 79% of males and 55% of females reporting it. Men reported hearing about HIV from more sources than women, with 50% of men reporting 3 or more sources, compared to 28% of women. All other sources of knowledge were more likely to be reported by men than women. HIV-positive people were more likely to report hearing about HIV from nearly every source compared to those who were HIV-negative, with the exception of school.

Figure 1 also shows the percentage of people who mentioned each mode of transmission, by sex and HIV status. The most commonly reported mode of transmission was through sexual intercourse. Women were most likely to report sex with high risk partners (48% of all women), whereas men were most likely to report unprotected sex as modes of transmission (56% of all men). Less than 4% of women reported knowing that HIV could be transmitted from mother to child.

### Experience of HIV and VCT

Table 3 shows the distribution of HIV and VCT experience by sex and HIV status. 51% of men and 45% of women answered at least one out of four questions affirmatively on knowing people living with HIV, or who had died of AIDS, with men and HIV-infected people being more likely to report knowing people with HIV.

**Table 3:** **HIV and VCT experience, by sex and HIV status**^**1**^

**Variable**	**Total**	**Male**		**Female**		**HIV negative**		**HIV positive**	
		**(n)**	**(%)**	**n**	**(%)**	**n**	**(%)**	**n**	**(%)**
**Total**	9, 461	3, 881	(41)	5, 575	(59)	8, 848	(94)	581	(6)
**HIV experience by sex and HIV status**									
Are any of your relatives HIV-infected?									
Yes	1, 501	531	(14)	970	(17)	1, 377	(16)	120	(21)
No	7, 951	3, 351	(86)	4, 600	(83)	7, 457	(84)	461	(79)
Have any of your relatives died of ADIS?									
Yes	1, 996	759	(20)	1, 237	(22)	1, 846	(21)	145	(25)
No	7, 456	3, 123	(80)	4, 333	(78)	6, 988	(79)	436	(75)
Is anyone in this village infected with HIV?									
Yes	3, 125	1, 482	(38)	1, 643	(29)	2, 886	(33)	225	(39)
No	6, 327	2, 400	(62)	3, 927	(71)	5, 948	(67)	356	(61)
Has anyone in the village died of AIDS?									
Yes	3, 647	1, 710	(44)	1, 937	(35)	3, 384	(38)	250	(43)
No	5, 805	2, 172	(56)	3, 633	(65)	5, 450	(62)	331	(57)
Have experience of HIV (composite indicator)									
Yes	4, 464	1, 970	(51)	2, 494	(45)	4, 150	(47)	298	(51)
No	4, 997	1, 916	(49)	3, 081	(55)	4, 698	(53)	283	(49)
**VCT experience &attitudes by sex and HIV status**									
Previously had VCT									
Yes	1, 178	554	(14)	624	(11)	1, 046	(12)	128	(22)
No	8, 724	3, 328	(86)	4, 964	(89)	7, 788	(88)	453	(78)
Would recommend VCT to a friend									
Yes	1, 101	518	(94)	583	(93)	981	(94)	118	(92)
No	77	36	(6)	41	(7)	65	(6)	10	(8)
VCT counsellor was kind and understanding									
Yes	1, 093	528	(95)	565	(91)	971	(93)	118	(92)
No	85	26	(5)	59	(9)	75	(7)	10	(8)
VCT interview was not difficult or embarrassing									
Yes	1, 106	524	(95)	582	(93)	984	(94)	118	(92)
No	71	30	(5)	41	(7)	61	(6)	10	(8)
VCT counsellors can be trusted to keep results secret									
Yes	921	483	(87)	438	(70)	815	(78)	105	(82)
No	256	70	(13)	186	(30)	230	(22)	23	(18)
If a person is seen going into a VCT centre people do not assume that he/she is infected									
Yes	737	372	(67)	365	(58)	658	(63)	77	(60)
No	440	181	(33)	259	(42)	387	(37)	51	(40)
Experience of VCT (composite indicator)									
Good	948	477	(12)	470	(8)	842	(10)	183	(18)
Poor	232	78	(2)	154	(3)	405	(2)	25	(4)
None	8, 278	3, 327	(86)	4, 946	(89)	7, 787	(88)	453	(78)

^1^All percentages in this table are column percentages for each variable except the first row of the table which gives the percentage of all (i.e. 41% respondents were male, 94% were HIV negatives).

Overall, 14% of men versus 11% of women and 15% of HIV-infected versus 12% of HIV-negatives had previously undergone VCT. The great majority of those who had previously had VCT (regardless of sex or HIV status) viewed it as a positive experience: over 90% would recommend it to a friend, thought the counsellor was kind and understanding, and that the interview was not difficult or embarrassing. Slightly lower proportions thought counsellors could be trusted to keep the results secret (87% of males, 70% of females). However, because such a low proportion of participants had any prior experience of VCT, only 12% of men and 8% of women overall could be classified as having had a good previous experience of VCT.

### Uptake of voluntary counselling and testing

749/3,886 men (19%) and 896/5,575 women (16%) used the VCT services offered during the survey (Table 3). Among men, VCT uptake was highest among those with good HIV knowledge, good ART knowledge, considerable experience of HIV and a good previous experience of VCT. It was also highest among those aged 25–34, those with more education, those who were HIV positive and those who had ever married. There was little difference in the rates of VCT use at the survey across rural and roadside villages and the trading centre (20%, 19% and 18% respectively). Among women, the patterns were broadly similar with the exception that VCT uptake was highest among those who had never married and among those living in the trading centre.

Table 4 shows the crude odds ratios for factors associated with uptake of VCT at the survey by sex. Among men, the crude odds of VCT uptake were lower among those with poor or fair HIV knowledge compared to those with good knowledge (chi-squared test for trend, p < 0.001) and lower among those with poor ART knowledge compared to those with good or fair ART knowledge (p < 0.001). The crude odds of VCT uptake were highest among those with considerable experience of HIV compared to those with either little or none (cOR = 1.7; 95% CI: 1.5-2.0), and were also higher among those with past VCT experience, whether this was described as poor (cOR = 2; 95% CI: 1.2-3.3) or good (cOR = 2.9; 95% CI: 2.4-3.6) compared to those who had never previously used VCT services. Other factors that were statistically significantly associated with VCT uptake were HIV status, age, education and marital status.

**Table 4:** Factors associated with VCT uptake – crude analysis

		**Men**			**Women**		
**Variables**	**Level**	**OR**	**(95% CI)**	**p-value**	**OR**	**(95% CI)**	**p-value**
HIV knowledge	Good	1			1		
	Fair	0.6	(0.5, 0.7)	<0.001	0.6	(0.5, 0.7)	<0.001
	Poor	0.3	(0.2, 0.5)	<0.001	0.3	(0.3, 0.5)	<0.001
ART knowledge	Good or fair	1			1		
	Poor	0.49	(0.4, 0.6)	<0.001	0.48	(0.4, 0.6)	<0.001
HIV experience	Yes	1.7	(1.5, 2.0)	<0.001	2.1	(1.8, 2.4)	<0.001
	No	1			1		
VCT experience	Good	2.9	(2.4, 3.6)	<0.001	2.7	(2.2, 3.3)	<0.001
	Poor	2	(1.2, 3.3)	0.007	2.5	(1.7, 3.5)	<0.001
	None	1			1		
HIV status	Negative	1			1		
	Positive	2	(1.4, 2.7)	<0.001	1.6	(1.2, 2.0)	<0.001
Age (years)	15-24	1			1		
	24-34	2.6	(2.1, 3.2)	<0.001	1.5	(1.3, 1.8)	<0.001
	35-44	2.2	(1.7, 2.8)	<0.001	1.3	(1.1, 1.7)	0.005
	45-54	1.9	(1.4, 2.5)	<0.001	1.0	(0.8, 1.3)	0.879
	55+	1.0	(0.7, 1.3)	0.839	0.2	(0.2, 0.4)	<0.001
Education	None	0.6	(0.5, 0.8)	<0.001	0.5	(0.5, 0.6)	<0.001
	Primary 1-4	0.7	(0.6, 0.9)	0.018	0.8	(0.6, 1.1)	0.171
	Primary 5-7	1			1		
	Secondary or above	1.4	(1.1, 1.7)	0.003	1.2	(1.0, 1.6)	0.087
Area of residence	Rural	1			1		
	Roadside	0.9	(0.8, 1.2)	0.548	1.0	(0.8, 1.2)	0.887
	Trading centre	0.9	(0.7, 1.1)	0.2	1.3		0.003
Marital status	Ever married	1.0			1	(1.1, 1.6)	
	Never married	0.8	(0.6, 0.9)	0.003	1.3	(1.0, 1.6)	0.042

Among women, the crude odds of VCT uptake were of a similar magnitude to those observed for men in terms of HIV knowledge, ART knowledge, HIV experience and VCT experience. The other statistically significant factors associated with VCT use at the survey among women were HIV status, age, education, residence and marital status.

Table 5 shows the adjusted odds of VCT use at the survey for men and women. Among men, a poor or fair HIV knowledge score reduced the odds of having VCT (aOR = 0.5; 95% CI: 0.3-0.9 and aOR = 0.8; 95% CI: 0.6-1.0 respectively). There was also borderline evidence of lower odds of VCT use among those with poor ART knowledge compared to those with fair or good ART knowledge (aOR = 0.8; 95% CI: 0.6-1.0). The odds of VCT were higher among those with considerable direct experience of HIV (aOR = 1.3; 95% CI: 1.1-1.5) and good past VCT experience (aOR = 2.0; 95% CI: 1.6-2.5), while there was borderline evidence that even a poor previous VCT experience was associated with reusing VCT at the survey (aOR = 1.5; 95% CI: 0.9-2.5). The odds of VCT uptake also remained independently associated with age, education and area of residence, and weakly associated with positive HIV status after controlling for the other potential confounders.

**Table 5:** **Factors associated with VCT uptake – adjusted analysis**^**1**^

		**Males**			**Females**		
**Variables**	**Category**	**aOR**	**95% CI**	**p-value**	**aOR**	**95% CI**	**p-value**
HIV knowledge	Good	1			1		
	Fair	0.8	(0.6, 1.0)	0.061	0.8	(0.7, 0.9)	0.004
	Poor	0.5	(0.3, 0.9)	0.014	0.6	(0.5, 0.9)	0.007
ART knowledge	Good or fair	1			1		
	Poor	0.8	(0.6, 1.0)	0.064	0.8	(0.6, 0.9)	0.003
HIV experience	Yes	1.3	(1.1, 1.5)	0.007	1.6	(1.4, 1.9)	<0.001
	No	1			1		
VCT experience	Good	1					
	Poor	2.0	(1.6, 2.5)	<0.001			
	None	1.5	(0.9, 2.5)	0.138			
Age (years)	15-24	1			1		
	25-34	2.3	(1.9, 2.9)	<0.001	1.3	(1.1, 1.6)	0.007
	35-44	1.9	(1.5, 2.5)	<0.001	1.2	(0.9, 1.4)	0.158
	45-54	1.7	(1.3, 2.3)	0.001	0.9	(0.7, 1.2)	0.571
	55+	1.1	(0.8, 1.5)	0.763	0.3	(0.2, 0.4)	<0.001
Education	None	0.8	(0.6, 1.0)	0.067			
	Primary 1-4	0.8	(0.6, 1.1)	0.202			
	Primary 5-7	1					
	Secondary	1.5	(1.2, 1.8)	0.002			
HIV status	Negative	1			1		
	Positive	1.4	(1.0, 1.9)	0.061	1.7	(1.3, 2.2)	<0.001
Area of residence	Rural	1					
	Peri-urban	0.9	(0.7, 1.1)	0.328			
	Urban	0.8	(0.6, 1.0)	0.024			
VCT experience in HIV negatives	Good experience				2.0	(1.6, 2.6)	<0.001
	Poor experience				2.1	(1.4, 3.1)	<0.001
	No experience				1		
VCT experience in HIV positives	Good experience				0.5	(0.3, 1.1)	0.090
	Poor experience				0.7	(0.2, 2.2)	0.521
	No experience				1		

^1^Adjusted for each of the other factors in the table.

Among women, very similar findings emerged in the adjusted analysis in terms of the magnitude of the effect of HIV and ART knowledge on VCT uptake. In terms of direct experience of HIV, women with considerable experience had 60% higher odds of using VCT at the survey compared to those with little or no direct experience of HIV (95% CI: 1.4-1.9). The effect of past VCT experience on VCT use was modified by HIV status: among HIV-positive women, there was borderline evidence that the odds of VCT uptake were 0.5 times lower among those with good prior VCT experience compared to those with no past VCT experience (95% CI: 0.3-1.1), and no evidence of a difference between those with poor past experience and no experience. Among HIV-negative women, the odds of VCT uptake were around twice as high for those with either good or poor previous VCT experience when compared to those who had never previously used VCT services (p < 0.001). HIV status remained associated with VCT uptake (aOR = 1.7 for HIV positive women compared to HIV negative women: 95% CI: 1.3-2.2), as did age, whereas there was no association between education, marital status or area of residence and VCT uptake in the adjusted analysis.

## Discussion

This study identified worryingly poor levels of knowledge about HIV transmission in a community that has been seriously affected by the epidemic over the course of the past two decades [16,17]. Furthermore, these highly prevalent misconceptions about HIV and ART occurred in a setting that has seen a variety of government and NGO-led HIV initiatives since 1995, including a school-based education programme, mapping of high risk areas for HIV transmission, condom promotion, the establishment of village AIDS committees to provide HIV education to the local community (since 2003), and a home-based care programme, PLHA support group and VCT and ART services (since 2005) [17,18].

In particular, although nearly all participants had heard about HIV, only 57% were deemed to have “good” HIV knowledge, with a considerable gap observed between the proportion of participants reporting to know how HIV is transmitted, and the proportion able to correctly identify three or more modes of transmission, which was only 28% among women. Particularly worrying was the extremely low proportion of all women who knew that HIV could be transmitted vertically. Subsequent analysis restricted to HIV-infected females of child-bearing age indicated that only 5% knew that HIV could be transmitted from mother to child. This shocking statistic is without doubt linked to the low levels of uptake of PMTCT observed in the region [19].

Not only did men tend to have more accurate knowledge about HIV transmission in this setting, but they also secured this knowledge from a far wider variety of sources compared to women, who tended to rely on family members, radio and to a lesser extent on health workers. Given the likely inaccuracies in HIV knowledge being transferred by family members in this setting, and the wide range of information available through radio programmes (including NGO-funded, government-supported messages but also advertising for evangelical churches), these results demonstrate the importance of ensuring that more accurate HIV information, education and communication is available to the community through a variety of different channels. For example, systematically informing women about HIV transmission and HIV-related services during health facility visits, including consultations they make for their children and their own antenatal appointments should be prioritised, and must go beyond the routine offer of a HIV test, in isolation from more comprehensive information about HIV and related services.

Similarly, overall, knowledge about ART was poor with only 16% of men, 10% of women and 18% of HIV-infected persons having good knowledge about its availability and use, despite the fact that the population had had access to the government’s free VCT and ART programme for around two years by the time of the survey. Furthermore, fear of the harmful effects of ART seems to be common among both men and women, supporting the findings of a qualitative study of barriers to sustaining ART in the same setting [8].

Existing structures, including the Village AIDS Committees, which were established in 2003 with the very purpose of providing accurate information to the community on HIV transmission and VCT/ART availability, could be better utilised in this setting to go some way to addressing these concerns. However, once established, these groups need ongoing and substantial support, including regular training, if they are to become a more widely recognised source of accurate information for the local community, particularly since previous research has shown that committee members themselves often hold inaccurate HIV and ART-related beliefs that can be disseminated through the community [10,18].

Only 19% of men and 16% of women took the opportunity to use the separate VCT service that was offered to them during this sero-survey. Although this is an increase of almost 60% and 130% respectively compared to the previous sero-survey in 2003-4 [20], it is much lower than the uptake of testing that has been reported from some other strategies, such as home-based counselling and testing [21]. However, further research is needed to explore whether this strategy would be feasible and acceptable in Tanzania, and whether it would result in effective links to HIV care and treatment for those testing positive.

The adjusted analysis indicated that accurate knowledge about HIV transmission and ART provision were both independently associated with VCT uptake for both men and women, with a similar magnitude of effect. Investments made in improving people’s accurate knowledge about HIV and ART are therefore likely to pay dividends in terms of increasing uptake of VCT services, which is an important step towards HIV-infected people accessing treatment and all clients receiving information about HIV and associated risk reduction strategies.

Direct experience of HIV was also significantly associated with having VCT at the survey for both men and women. Previous reports from this setting have indicated that seeing someone suffering with AIDS, or recovering because of ART encourages others to get tested in order to potentially access treatment [8]. In addition, knowing someone with HIV may help people to see that they are not different, and to become aware that they may also be at risk of HIV. Either way, increasing people’s contact with people living with HIV could help to increase community-level uptake of VCT services, if done in a culturally-appropriate manner. This could be achieved through having ‘expert patients’ talking openly about their status in churches or other public meetings, or by encouraging well-respected village leaders to visibly undergo HIV testing, although high levels of HIV-related stigma in some rural settings may make this challenging.

The findings from this study need to be considered in light of several limitations. Although the survey had a large sample size (n = 9,461), only 61% of eligible adults participated [15], resulting in possible selection bias, if those who participated were systematically different to those who did not in relation to the distribution of the key variables under investigation. For example, previous analyses have indicated that non-participation is highest among males living in roadside villages and the trading centre [15]. These individuals tend to have more accurate HIV and ART knowledge, but similar levels of VCT uptake compared to those in the more rural villages, suggesting that their relatively lower participation may lead to a slight underestimate of overall ART and HIV knowledge levels in this setting, but would not affect overall estimates of VCT uptake, nor the observed associations between HIV/ART knowledge and use of VCT services. An additional issue relates to social desirability bias in relation to the survey questions that rely on self-report on sensitive topics including previous VCT use and direct experience of HIV. Finally, the data used in this analysis were collected in 2006–7, and it is likely that levels of HIV and ART knowledge as well as motivation to use VCT services have since increased. Nevertheless, these analyses form an important baseline against which to compare trends in HIV and ART knowledge and their association with VCT use over time, as additional survey rounds are conducted.

The strengths of this study lie in its ability to link data on HIV and ART knowledge to information on actual VCT use during the survey and to HIV status among all participants, regardless of whether they had used VCT services or not. Furthermore, as the sample covers the general population of adults residing within the study area, the findings are likely to be generalisable to other rural settings in Tanzania and possibly beyond.

## Conclusion

In conclusion, misconceptions around HIV transmission and ART provision remain rife in this setting despite the availability of free HIV testing and treatment services. Furthermore, the uptake of VCT services remains low, even when they are provided at the village level and transportation barriers are thus removed. Inaccurate knowledge about HIV and ART is associated with non-use of VCT services, and urgently needs to be addressed through a variety of rigorously tested information, education and communication strategies.

## Competing interests

The authors declare that they have no competing interests.

## Authors’ contributions

AS carried out the analysis and wrote the first draft of the manuscript. AW contributed to the analysis, the interpretation of the results and the writing of the manuscript. YK and RI participated in the collection and management of the serosurvey data. RM participated in the collection of the HIV testing data. CC participated in the data management and contributed to writing the manuscript. MU and JT were responsible for the implementation and overall coordination of the study and for overseeing the data collection and management. BZ conceived of the study and its design. All authors read and approved the final manuscript.

## Pre-publication history

The pre-publication history for this paper can be accessed here:

http://www.biomedcentral.com/1471-2458/13/802/prepub
